# Splanchnic Circulation and Intraabdominal Metabolism in Two Porcine Models of Low Cardiac Output

**DOI:** 10.1007/s12265-018-9845-6

**Published:** 2018-11-19

**Authors:** Jenny Seilitz, Tal M. Hörer, Per Skoog, Mitra Sadeghi, Kjell Jansson, Birger Axelsson, Kristofer F. Nilsson

**Affiliations:** 1 Department of Cardiothoracic and Vascular Surgery, Faculty of Medicine and Health, Örebro University Hospital, SE-70185 Örebro, Sweden; 2 Department of Vascular Surgery and Institute of Medicine, Department of Molecular and Clinical Medicine, Sahlgrenska University Hospital and Academy, Gothenburg, Sweden; 3 Department of Vascular Surgery, Västmanland’s Hospital, Västerås, Sweden and Faculty of Medicine and Health, Örebro University, Örebro, Sweden; 4 Department of Surgery, Faculty of Medicine and Health, Örebro University, Örebro, Sweden

**Keywords:** Cardiac dysfunction, Cardiac tamponade, Caval vein balloon, Porcine model, Intraperitoneal microdialysis, Laser Doppler flowmetry

## Abstract

The impact of acute cardiac dysfunction on the gastrointestinal tract was investigated in anesthetized and instrumented pigs by sequential reductions of cardiac output (CO). Using a cardiac tamponade (*n* = 6) or partial inferior caval vein balloon inflation (*n* = 6), CO was controllably reduced for 1 h each to 75% (CO_75%_), 50% (CO_50%_), and 35% (CO_35%_) of the baseline value. Cardiac output in controls (*n* = 6) was not manipulated and maintained. Mean arterial pressure, superior mesenteric arterial blood flow, and intestinal mucosal perfusion started to decrease at CO_50%_ in the intervention groups. The decrease in superior mesenteric arterial blood flow was non-linear and exaggerated at CO_35%_. Systemic, venous mesenteric, and intraperitoneal lactate concentrations increased in the intervention groups from CO_50%_. Global and mesenteric oxygen uptake decreased at CO_35%_. In conclusion, gastrointestinal metabolism became increasingly anaerobic when CO was reduced by 50%. Anaerobic gastrointestinal metabolism in low CO can be detected using intraperitoneal microdialysis.

## Introduction

Critically ill patients and patients undergoing cardiac surgery frequently develop cardiac dysfunction with low cardiac output (CO) [1, 2]. Low CO causes impairment of splanchnic circulation, leading to loss of gastrointestinal (GI) mucosal barrier function and bacterial translocation, which may initiate, maintain, or aggravate a systemic inflammatory reaction [3]. These processes may progress to GI complications, which include GI bleeding, mesenteric ischemia, and pancreatitis [4–6].

From human studies, it is difficult to establish which degree of CO reduction impairs GI metabolism, and in a clinical setting, this would be difficult or even impossible to investigate in a well-regulated manner. Previous animal studies using different methods of splanchnic blood flow reduction, however not limited to low cardiac output, have found that approximately a halving or more of GI blood flow induced a GI metabolic disorder, including decreased mucosal oxygen tension, mucosal pH and mesenteric oxygen uptake, and increased jejunal mucosal-to-arterial PCO_2_ gradient [7–9]. Further metabolic consequences were increased mesenteric venous-arterial lactate difference and ß-hydroxybutyrate/acetoacetate ratio as well as increased intestinal intraluminal and intramural lactate concentrations and lactate/pyruvate ratios [7, 10]. The specific methods for detection of splanchnic anaerobic metabolism, e.g., intestinal intraluminal and intramural microdialysis, jejunal tonometry, and jejunal mucosal oxygen tension, used in these studies are predominantly applicable in experimental investigations. In contrast, percutaneous insertion of intraperitoneal microdialysis catheters is possible in non-abdominal surgical patients, e.g., in endovascular surgery of ruptured aortic aneurysm and critical care patients [11, 12]. A pilot study of intraperitoneal microdialysis with measurements of lactate, pyruvate, glucose, and glycerol showed its feasibility in cardiac surgical patients [13], whereas it has not been investigated in low cardiac output models.

Several experimental models for creating acute cardiac dysfunction and low CO by myocardial injury have been described, including percutaneous injections of microspheres [14] or ethanol in a coronary artery [15], percutaneous balloon occlusion [16], or ligation of one or several coronary arteries [17–19]. Although clinically relevant, one disadvantage of these models is that CO cannot easily be titrated to a preset level. Models of GI in-flow restriction are easily adjustable, but if the GI tract is the target organ of interest, the splanchnic venous outflow pressure must be increased [20]. Obstruction of right heart filling by an adjustable cardiac tamponade [7, 21] and the reduction of cardiac preload by variable inflation of an inferior caval vein balloon [22, 23] are adjustable low cardiac output models with potentially mesenteric venous congestion.

Therefore, in the current study, we used these two porcine models—cardiac tamponade and caval vein balloon inflation—and investigated the impact of low CO on the splanchnic circulation and metabolism including intraperitoneal microdialysis. We hypothesized that a 50% reduction in CO causes an impairment of the GI circulation and metabolism detectable with intraperitoneal microdialysis.

## Methods

### Animals

The experiments were approved by the regional animal ethics committee and were conducted in accordance with the directive of the European Union for the protection of animals used for scientific purposes [24]. In this study, 24 healthy 3-month-old domestic pigs (a crossbreed between Swedish country breed, Hampshire and Yorkshire) of both sexes and with a median body weight of 30 kg (range 25–41 kg) were used.

### Anesthesia, Fluid Administration, Ventilation, and Euthanasia

On the farm, the animals were given azaperone (200 mg, i.m.; Elanco, Herlev, Denmark). On arrival at the research facility, anesthesia was induced by tiletamine (6 mg kg^−1^, i.m.; Virbac, Kolding, Denmark), zolazepam (6 mg kg^−1^, i.m.; Virbac), and azaperone i.m. (4 mg kg^−1^). Atropine (1.5 mg, i.m.; Mylan, Stockholm, Sweden) was given. Two peripheral catheters (1.1 mm, Venflon™ Pro Safety, BD, Helsingborg, Sweden) were inserted in auricular veins. Propofol (1–2 mg kg^−1^, i.v.; Fresenius Kabi, Uppsala, Sweden) was given if needed. The pigs were orally intubated with a 6-mm endotracheal tube (Covidien, Tullamore, Ireland). Anesthesia was maintained with propofol (10–15 mg kg^−1^ h^−1^, i.v.) and fentanyl (5–20 μg kg^−1^ h^−1^, i.v; Meda, Solna, Sweden) given by motorized syringe pumps (Alaris CC, Cardinal Health, Rulle, Switzerland). The depth of anesthesia was intermittently checked by pain response. Cefuroxim (750 mg, i.v.; GSK, Solna, Sweden) was given before, and heparin (5000 IU, i.v.; LEO Pharma, Malmö, Sweden) after instrumentation. Ringer’s acetate (10 ml kg^−1^ h^−1^, i.v.; Fresenius Kabi) and 10% glucose with 40 mM sodium and 20 mM potassium (0.5 ml kg^−1^ h^−1^, i.v.; Fresenius Kabi) were administered, by volume pumps (Alaris GP, CareFusion). The pigs were ventilated in volume control ventilation mode (PV 501, Breas Medical AB, Sweden) to achieve arterial PCO_2_ of 4.8–5.5 kPa, and the fraction of inspired O_2_ was adjusted to maintain arterial PO_2_ at 11–18 kPa. The positive-end expiratory pressure was set at 4–6 cm H_2_O. Respiratory variables and gases were measured at the endotracheal tube (AS/3, Datex, Helsinki, Finland). The body temperature was kept at 37.5–38.5 °C by using a thermal mattress and a forced-air warming blanket. At the end of the experiment, the anesthetized animals were euthanized by a rapid i.v. injection of 40 mmol potassium chloride (B. Braun, Danderyd, Sweden), and asystole and circulatory arrest were confirmed by ECG and blood pressure recordings.

### Surgical Preparation and Measurements

In the left external jugular vein, a 7-Fr triple-lumen central line (Arrow/Vingmed, Järfälla, Sweden) was inserted for fluid and drug administration. In the right carotid artery, a 4-Fr introducer (Cordis Corporation, Miami Lakes, FL, USA) was positioned for measurement of systemic blood pressure and heart rate and also for blood sampling. In the right external jugular vein, an 8-Fr introducer was inserted (Cordis Corporation). Through this, a pulmonary artery catheter (Swan-Ganz CCOmbo, 7.5 Fr, Edwards Lifesciences, Irvine, CA, USA) was positioned, with its tip in a branch of the pulmonary artery, under the guidance of the pressure curve. In addition, correct placement was confirmed by fluoroscopy (Philips BV 300, Stockholm, Sweden). The pulmonary artery catheter was used for blood sampling and measurements of pulmonary capillary wedge pressure (PCWP), central venous pressure (CVP), and CO using the semi-continuous thermodilution technique (Vigilance, Edwards Lifesciences).

A midline abdominal incision was performed. A 14-Fr Foley catheter (Unomedical, Flintshire, UK) was inserted in the bladder and fixed with a purse string suture. A laser Doppler probe (Probe 427, Perimed, Järfälla, Sweden), connected to a laser Doppler device (Periflux System 5000, Perimed), was placed intraluminally, pointing toward the intestinal mucosa in a mid-jejunal intestinal loop approximately 30 cm distal to the Treitz ligament for estimation of local mucosal perfusion [25]. An ultrasonic transit-time flowmeter (Vascular TTFM Probe 6 mm, Medistim ASA, Oslo, Norway) was placed around the superior mesenteric artery (SMA). A 6-Fr catheter (Nutrisafe 2, Vygon, Ecouen, France) was inserted in a distal branch and advanced to the superior mesenteric vein for pressure measurement and blood sampling. The blood samples were analyzed for pH, blood gases, electrolytes, lactate, hemoglobin, and glucose, using either i-STAT (Abbott Scandinavia, Solna, Sweden) or GEM Premier 4000 (Instrumentation Laboratory, Lexington, MA, USA), at 37 °C. Blood pressures were measured by a pressure transducer (Codan, Forstinnigen, Germany) connected to the AS/3 (Datex). A free-floating microdialysis catheter (62 Gastrointestinal Microdialysis Catheter, membrane length 30 mm, cut-off 20 kDa, M Dialysis, Stockholm, Sweden) was placed intraperitoneally in the left lower quadrant [26]. A syringe pump (107 Microdialysis Pump, M Dialysis) was used to propel a solution (Perfusion fluid T1, M Dialysis) at 2 μL min^−1^ through the microdialysis catheter. The microdialysate was analyzed for glucose, glycerol, lactate, and pyruvate (CMA 600, M Dialysis).

### Specific Preparation of Animals in the Cardiac Tamponade and Control Groups

In the control and the tamponade groups, a 12-Fr Foley catheter (Unomedical) was inserted into the pericardial space via a small incision through a diaphragmatic window and sutured in place. Subcutaneous fat was used as pledgets to reduce the risk of leakage. Approximately 5 ml of physiological sodium chloride solution (B. Braun) was instilled in the balloon of the Foley catheter.

### Specific Preparation of Animals in the Inferior Caval Vein Balloon Group

In the animals allocated to the caval vein balloon group, an additional 11-Fr introducer (Cordis Corporation) was inserted in the right external jugular vein. A balloon catheter (Cordis PTA Dilatation Catheter, large diameter balloon, 7 Fr, 80 cm, Cordis Corporation) was advanced to the diaphragmatic part of the inferior caval vein under fluoroscopic guidance.

### Protocol

After an intervention-free period of 1 h to achieve stable baseline conditions, the animals were divided into three groups: (1) the tamponade group, (2) the caval vein balloon group, and (3) the control group. In the tamponade group, CO was reduced by instillation of a colloid fluid (at 38 °C; 6% hydroxyethyl starch, Fresenius Kabi) via the pericardial Foley catheter into the closed pericardium. Cardiac tamponade was confirmed by transthoracic echocardiography (SonoSite Titan™, L52/10–5 MHz Transducer, Askim, Sweden). In the caval vein balloon group, CO was decreased by partial inflation of the balloon. Inflation of the balloon was confirmed by fluoroscopy. After collecting baseline values, blood samples, and microdialysate, CO was stepwise lowered in these groups to 75% (CO_75%_), 50% (CO_50%_), and 35% (CO_35%_) of baseline value for 1 h each, at which point respiratory and hemodynamic variables as well as blood samples were collected. The microdialysate was gathered during the last 30 min at each CO level. In the control group, no intervention was made to affect CO, but data collection was identical to that of the two other groups.

### Calculations

Cardiac output and SMA blood flow were divided by body surface area, according to the formula: body surface area (m^2^) = 0.0734 × weight (kg)^0.656^, to obtain the cardiac index (CI) and the SMA blood flow index [27]. Oxygen saturation was calculated as PO_2_^2.94^/(PO_2_^2.94^ + *P*_50_^2.94^). 4.76 kPa was used as the porcine partial pressure of oxygen (PO_2_), where hemoglobin is half-saturated (*P*_50_), and the value was adjusted with the fixed acid Bohr coefficient [28]. Oxygen content was calculated as saturation (fraction) × Hb (g/l) × 1.34 + 0.225 × PO_2_ (kPa) [29]. Oxygen extraction was calculated as arterial oxygen content subtracted by venous oxygen content. Oxygen delivery (DO_2_) was acquired as the blood flow index times arterial oxygen content, and oxygen uptake (VO_2_) was calculated as oxygen extraction times the blood flow index.

### Statistical Analysis

Data are presented as medians with interquartile ranges. In the statistical analysis, the absolute value was used at baseline, and thereafter, the difference or percentage change from baseline was used. The Bonferroni corrected Kruskal-Wallis tests were performed to identify statistical differences between groups at each CO level, and, if significant, they were followed by pairwise comparisons between groups using the Bonferroni corrected Mann-Whitney *U* tests. Least-squares linear and logarithmic regressions were made. A *P* value of less than 0.05 was regarded as statistically significant. Statistical analysis was performed using IBM SPSS Statistics version 22.0 for Windows (SPSS Inc., Chicago, IL, USA).

## Results

### Animal Inclusion

**Fig. 1:**
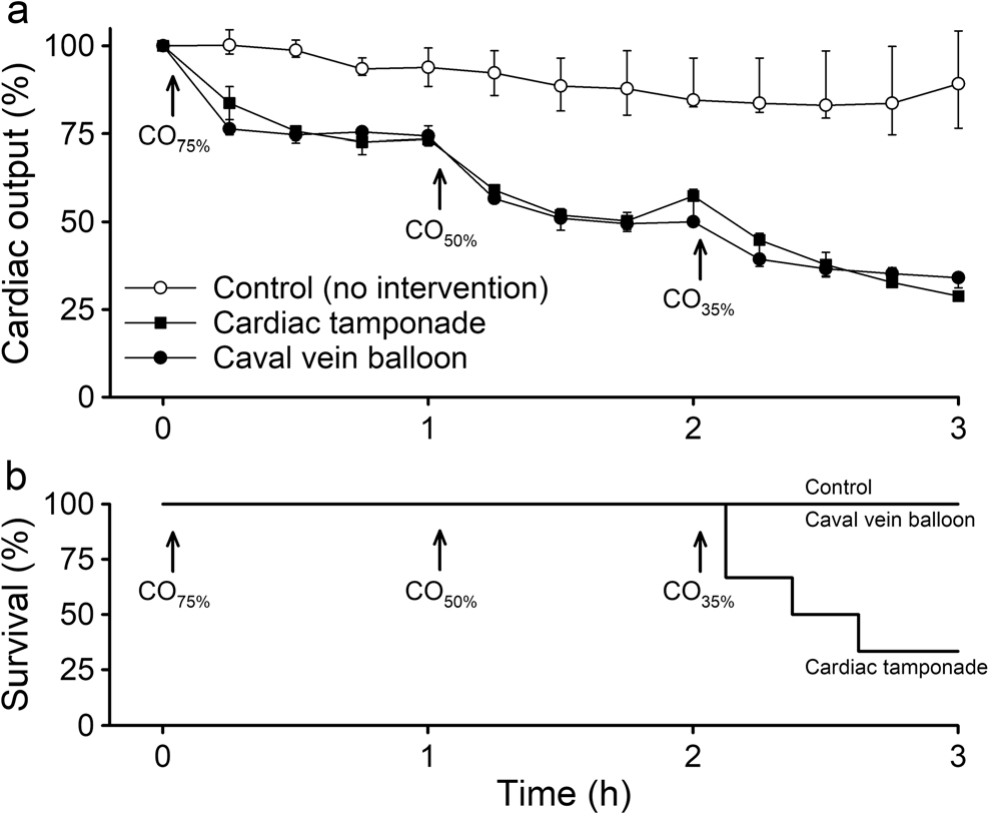
Cardiac output (CO, panel **a**, data are presented as medians and interquartile ranges) and survival (panel **b**) in anesthetized and mechanically ventilated pigs subjected to a graded reduction of CO to 75% (CO_75%_), 50% (CO_50%_), and 35% (CO_35%_) of the baseline value by either cardiac tamponade (*n* = 6) or partial inflation of an inferior caval vein balloon (*n* = 6). In one group, no intervention was made (control, *n* = 6)

Of the 24 animals used in the study, one pig was excluded due to circulatory instability after instrumentation, and one died suddenly during instrumentation. Nine animals were allocated to the tamponade group; two were excluded because of leakage from the pericardial sutures that could not be repaired, and one was excluded due to failure to collect microdialysate. Four of the six animals in the tamponade group died at CO_35%_ and were only included in the statistical analyses until CO_50%_ (Fig. 1b). Seven pigs were allocated to the caval vein balloon group; one died of an arrhythmia at CO_75%_ and was excluded. Six pigs were included in the control group.

### Cardiac Output

At baseline, CI values were 6.4 (5.5–7.5), 5.9 (4.8–6.6), and 6.7 (6.0–7.5) l min^−1^ m^−2^ in the control (*n* = 6), tamponade (*n* = 6), and caval vein balloon (*n* = 6) groups, respectively. Both inflation of the caval vein balloon and induction of the cardiac tamponade caused a controllable and steady lowering of CO to the targeted level, whereas the controls maintained their CO level throughout the study (Fig. 1a). After the desired level of CO was reached, no major adjustments of the pericardial volume or caval vein balloon inflation were required (data not presented).

### Central Hemodynamic Variables

**Fig. 2:**
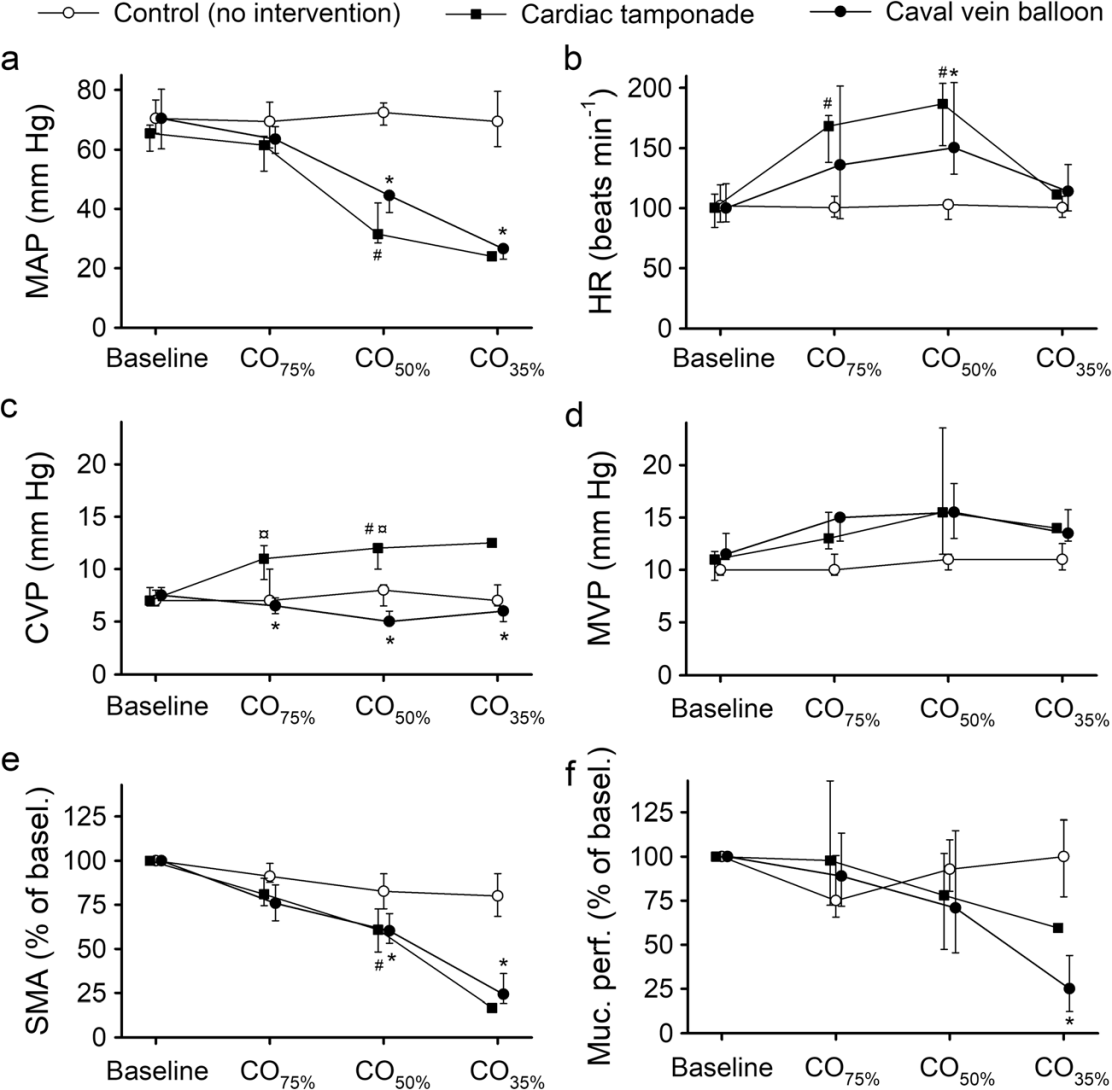
Mean arterial pressure (MAP, panel **a**), heart rate (HR, panel **b**), central venous pressure (CVP, panel **c**), mesenteric venous pressure (MVP, panel **d**), superior mesenteric arterial blood flow (SMA, panel **e**), and intestinal mucosal perfusion (Muc. perf., panel **f**) in anesthetized and mechanically ventilated pigs subjected to a graded reduction of cardiac output to 75% (CO_75%_), 50% (CO_50%_), and 35% (CO_35%_) of the baseline value by either cardiac tamponade (*n* = 6) or partial inflation of an inferior caval vein balloon (*n* = 6). In one group, no intervention was made (control, *n* = 6). Data are presented as medians and interquartile ranges. Asterisk, number, and currency symbols indicate a statistical difference (*P* < 0.05) between the caval vein balloon group and the control group, the cardiac tamponade group and the control group, and the caval vein balloon and cardiac tamponade groups, respectively

At CO_75%_, the mean arterial pressure (MAP) was unchanged in the tamponade and caval vein groups, compared to the control group (Fig. 2a). In both intervention groups, MAP decreased significantly when CO was further reduced (*P* < 0.05 compared to the control group, Fig. 2a). Heart rate followed a biphasic response in both intervention groups (Fig. 2b). CVP increased in the tamponade group and decreased in the caval vein group (*P* < 0.05 in both groups compared to the control group), while the mesenteric venous pressure (MVP) tended to increase in both intervention groups (Fig. 2c, d).

### Gastrointestinal Circulation

At baseline, SMA flow index values were 1.03 (0.90–1.42), 0.85 (0.60–1.29) and 1.04 (0.92–1.40) l min^−1^ m^−2^ in the control, tamponade, and caval vein balloon groups, respectively. SMA blood flow decreased significantly in both intervention groups as CI decreased (*P* < 0.05 compared to the control group, Fig. 2e). Intestinal mucosal perfusion was decreased at CO_35%_ (*P* < 0.05 for caval vein balloon versus controls, Fig. 2f).

### Relationship Between Cardiac Index and Blood Flow in the Superior Mesenteric Artery

**Fig. 3:**
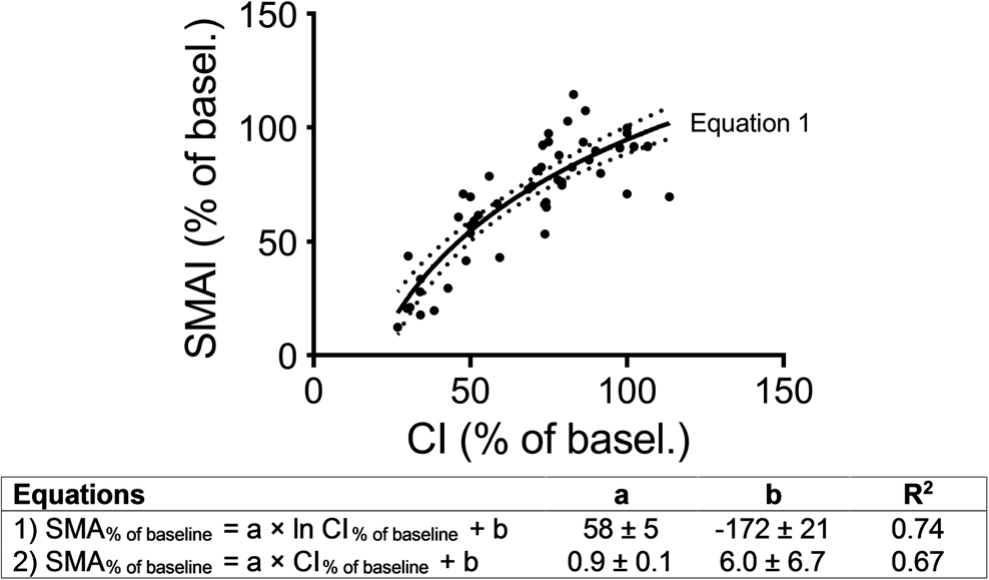
Curve estimations for the blood flow index in the superior mesenteric artery (SMAI) and the cardiac index (CI) in anesthetized and mechanically ventilated pigs subjected to a graded reduction of cardiac output by cardiac tamponade or partial inflation of an inferior caval vein balloon and controls

The decrease in SMA flow in the intervention groups followed the decrease in CI, but the SMA flow was higher than predicted at CO_50%_ (61% [48–73] and 60% [53–70]) in the tamponade and caval vein groups, respectively) and lower at CO_35%_ (17% and 24% [19–36] in the tamponade and caval vein groups, respectively; Figs. 2e and 3). To further explore the relationship between CI and SMA flow, linear and logarithmic regressions were carried out. Using SMA flow (percentage of baseline) as the dependent variable and CI (percentage of baseline) as the independent variable, regression analysis resulted in a better fit when using a natural logarithmic rather than a linear equation (Fig. 3).

### Respiratory Variables

Arterial PO_2_ and PCO_2_ and also minute ventilation and fraction of inspired O_2_ were normal, stable, and similar in all groups throughout the study (Table 1).

**Table 1:** Variables at baseline and after reduction of cardiac output in anesthetized pigs

	Baseline	CO_75%_	CO_50%_	CO_35%_
Arterial PO_2_ (kPa)				
Cardiac tamponade	12.9 (11.5–14.5)	12.9 (10.7–16.8)	15.0 (10.7–17.5)	10.2
Caval vein balloon	16.6 (14.9–18.9)	17.1 (16.4–18.1)	17.1 (16.8–19.5)	16.5 (15.9–19.8)
Control	13.6 (9.6–17.6)	16.8 (14.0–18.6)	16.0 (12.4–18.5)	15.0 (12.4–17.5)
Arterial PCO_2_ (kPa)				
Cardiac tamponade	5.0 (4.7–5.3)	5.0 (4.5–5.4)	4.6 (4.2–5.1)	4.4
Caval vein balloon	4.9 (4.8–5.4)	4.9 (4.7–5.2)	4.6 (4.3–5.3)	4.1 (3.5–4.5)
Control	4.9 (4.7–5.2)	4.7 (4.0–5.0)	4.8 (4.5–4.9)	4.6 (4.0–4.6)
Arterial pH				
Cardiac tamponade	7.51 (7.49–7.55)	7.50 (7.42–7.54)	7.42 (7.33–7.49)^#^	7.25
Caval vein balloon	7.50 (7.46–7.53)	7.50 (7.46–7.53)	7.44 (7.32–7.47)*	7.26 (7.22–7.37)*
Control	7.55 (7.50–7.56)	7.58 (7.55–7.63)	7.58 (7.54–7.60)	7.59 (7.75–7.61)
Arterial to mesenteric venous difference in lactate concentration (mM)				
Cardiac tamponade	− 0.07 (− 0.20–0.01)	0.06 (−0.16–0.34)	− 0.56 (− 1.07–0.25)	− 1.28
Caval vein balloon	− 0.05 (− 0.33–0.05)	− 0.15 (− 0.35–0.10)	− 0.60 (− 1.83–0.23)	− 2.15 (− 3.68–− 1.03)
Control	− 0.01 (− 0.30–0.08)	− 0.12 (− 0.30–0.10)	− 0.08 (− 0.18–0.02)	− 0.28 (− 0.49–− 0.10)
Intraperitoneal lactate (mM)				
Cardiac tamponade	5.2 (4.9–6.6)	7.5 (5.6–8.9)	10.0 (8.2–12.1)^#^	12.0
Caval vein balloon	3.6 (2.8–4.9)	4.7 (3.5–5.4)	5.8 (4.3–8.9)*	10.1 (7.8–14.6)*
Control	4.2 (3.2–5.3)	4.0 (3.2–6.2)	3.6 (3.0–5.2)	3.5 (2.9–6.8)
Intraperitoneal pyruvate (μM)				
Cardiac tamponade	297 (261–334)	327 (297–355)	389 (338–472)	399
Caval vein balloon	252 (151–395)	275 (219–372)	307 (269–393)	288 (241–447)
Control	346 (189–411)	338 (194–450)	318 (202–404)	302 (212–374)
Intraperitoneal lactate/pyruvate ratio				
Cardiac tamponade	18 (17–22)	23 (17–27)	25 (21–30)^#^	32
Caval vein balloon	14 (12–22)	16 (13–19)	17 (15–24)	33 (31–37)*
Control	15 (11–17)	13 (10–20)	13 (11–17)	13 (12–19)
Intraperitoneal glycerol (μM)				
Cardiac tamponade	101 (70–137)	89 (80–128)	123 (66–170)	173
Caval vein balloon	66 (52–211)	72 (58–114)	83 (77–149)	165 (130–365)*
Control	61 (47–90)	45 (32–83)	44 (36–58)	34 (29–50)
Intraperitoneal glucose (mM)				
Cardiac tamponade	4.2 (3.0–5-5)	3.5 (2.9–3.9)	2.6 (1.8–4.1)	3.0
Caval vein balloon	2.9 (1.6–3.6)	2.9 (2.0–3.9)	1.8 (1.5–2.2)	1.7 (0.6–3.6)
Control	4.4 (3.2–5.1)	3.8 (3.2–4.4)	3.7 (3.3–4.0)	2.8 (2.1–3.2)
Plasma glucose (mM)				
Cardiac tamponade	5.8 (5.7–5.9)^#^	6.0 (5.4–6.4)	6.3 (5.6–7.6)^#^	8.5
Caval vein balloon	5.3 (4.8–5.7)*	5.2 (4.9–5.7)	5.4 (4.8–7.2)*	5.4 (4.0–7.1)
Control	6.2 (6.0–6.9)	5.5 (5.4–6.0)	5.0 (4.7–5.5)	4.9 (4.4–5.2)

Data are presented as medians (interquartile ranges)

*n* = 6/group except at CO_35%_ in the cardiac tamponade group (*n* = 2)

*P < 0.05 control vs. caval vein balloon group

^#^*P* < 0.05 control vs. cardiac tamponade group

### Oxygen Indices

**Fig. 4:**
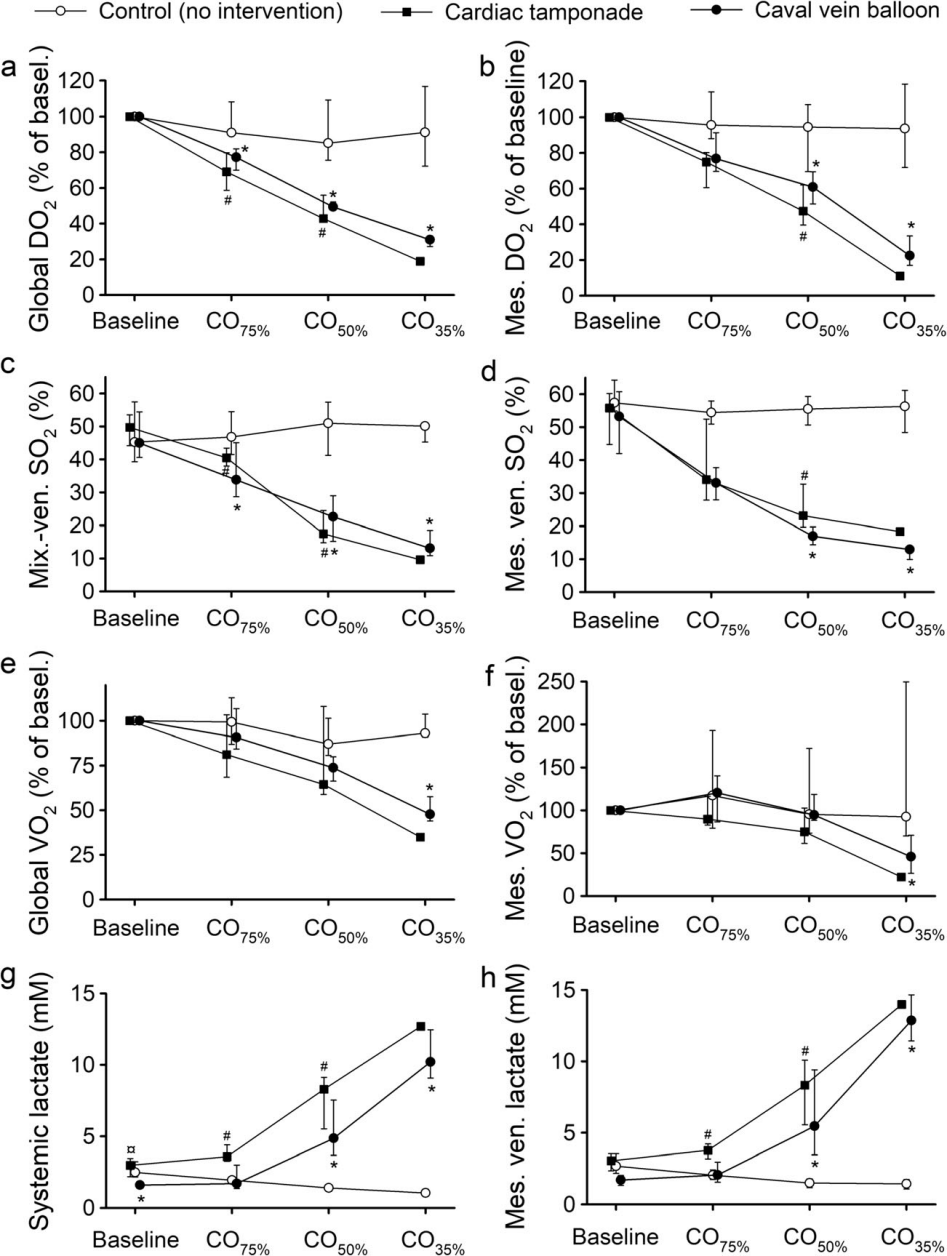
Global and mesenteric oxygen deliveries (DO_2_, panela **a** and **b**, respectively), mixed-venous saturation (Mix.-ven. SO_2_, panel **c**), mesenteric venous saturation (Mes. ven. SO_2_, panel **d**), global and mesenteric oxygen uptakes (VO_2_, panels **e** and **f**, respectively), and also systemic and mesenteric venous lactate concentrations (panels **g** and **h**, respectively) in anesthetized and mechanically ventilated pigs subjected to a graded reduction of cardiac output to 75% (CO_75%_), 50% (CO_50%_), and 35% (CO_35%_) of the baseline value by either cardiac tamponade (*n* = 6) or partial inflation of an inferior caval vein balloon (*n* = 6). In one group, no intervention was made (control, *n* = 6). Data are presented as medians and interquartile ranges. Asterisk, number, and currency symbols indicate a statistical difference (*P* < 0.05) between the caval vein balloon group and the control group, the cardiac tamponade group and the control group, and the caval vein balloon and cardiac tamponade groups, respectively

At baseline, systemic DO_2_ values were 550 (490–690), 690 (590–820), and 730 (590–810) ml O_2_ min^−1^ m^−2^ in the control, tamponade, and caval vein balloon groups, respectively, while mesenteric DO_2_ were 92 (75–138), 108 (62–170), and 116 (103–131) ml O_2_ min^−1^ m^−2^ in the respective groups. Systemic respective mesenteric VO_2_ were 300 (260–300) and 38 (27–59) ml O_2_ min^−1^ m^−2^ in the control group, 350 (250–430) and 47 (34–61) ml O_2_ min^−1^ m^−2^ in the tamponade group, and 360 (330–390) and 50 (46–68) ml O_2_ min^−1^ m^−2^ in the caval vein balloon group. Systemic and mesenteric DO_2_ decreased significantly when CO was reduced (*P* < 0.05 compared to control, Fig. 4a, b). In parallel, the mixed venous and mesenteric venous SO_2_ decreased significantly to very low values at CO_35%_ (*P* < 0.05 compared to controls, Fig. 4c, d). Significant decrements in systemic and mesenteric VO_2_ developed at CO_35%_ (*P* < 0.05, compared to controls, Fig. 4e, f).

### Metabolic Variables

Arterial pH decreased in the intervention groups and was significantly lower compared to the control group (*P* < 0.05) at CO_50%_ and CO_35%_ (Table 1). Arterial and mesenteric lactate levels increased significantly from CO_75%_ and CO_50%_ in the tamponade and caval vein balloon groups, respectively (*P* < 0.05 compared to the control group, Fig. 4g, h). The arterial-mesenteric venous differences of lactate tended to become more negative as CO was progressively reduced (Table 1). Intraperitoneal lactate concentrations increased significantly in both intervention groups and were significantly higher compared to the control group from CO_50%_ (*P* < 0.05, Table 1). Intraperitoneal lactate/pyruvate ratios increased from CO_50%_ and CO_35%_ in the tamponade and caval vein balloon groups, respectively (*P* < 0.05 compared to the control group, Table 1). Intraperitoneal glycerol levels increased at CO_35%_ in the caval vein group (*P* < 0.05 compared to the control group, Table 1). Intraperitoneal pyruvate and glucose concentrations and plasma glucose concentrations did not change significantly (Table 1).

## Discussion

In this study, two different methods to induce low CO states were used with the aim of simulating acute cardiac dysfunction. Important features of the present methods include a high degree of adjustability to a predetermined level of CO and the ability to maintain a stable CO after reaching target values. During the conduct of the experiments, the authors found the cardiac tamponade model more surgically demanding and that the responses to the titrations of CO in that model were less predictable. A noteworthy difference between the models was that only a minority of the animals in the cardiac tamponade group survived 1 h at the lowest CO level, which is in line with a previous study [7]. Both models compromise ventricular filling but with different cardiac pathology. Both methods induce preload-dependent systolic dysfunction, while cardiac tamponade also deteriorates diastolic function. The diastolic dysfunction composes an early diastolic collapse of the right ventricle, a late diastolic collapse of the right atrium, and an abnormal septal motion [30]. The increased pericardial pressure and the decreased MAP in cardiac tamponade may lower the myocardial blood flow resulting in myocardial ischemia [31]. The distinctive effects on cardiac function by the cardiac tamponade could explain the higher mortality at the very low CO levels. These differences in features between the models favor the use of the caval vein balloon model in future work aimed at studying distal organ function in low CO conditions.

In contrast to models of low CO induced by cardiac ischemia [14, 16, 17, 19, 32], neither of the presented models mirrors cardiac dysfunction with load-independent decreased contractility, which would have been a disadvantage if the cardiac function per se was the subject matter. However, from the perspective of the intestines, both mimic a combined backward and forward cardiac failure, which makes the models highly clinically relevant for gastrointestinal consequences of cardiac dysfunction.

Backward failure was indicated by raised MVP in both conditions. Forward failure caused decreases in SMA flow and also global and mesenteric DO_2_. MAP and intestinal mucosal perfusion were maintained at CO_75%_ and decreased thereafter. An effect on the metabolism was apparent first at CO_50%_ and was clearly pronounced at CO_35%_. VO_2_ decreased at CO_35%_ possibly due to reaching the limit of oxygen extraction capability when mesenteric venous SO_2_ entered the lower flat part of the hemoglobin oxygen dissociation curve.

In the present models, a global anaerobic state was induced. The GI tract contributed partly to this response, since the arterial-mesenteric venous lactate difference tended to become gradually more negative when reducing CO in parallel with increments in intraperitoneal lactate and glycerol concentrations. This is further supported by the decrease in intestinal VO_2_ at CO_35%_.

The present data indicate that the GI tract can withstand only a mild compromised circulation with major metabolic derangements evident at CO_50%_. These findings are in accordance with several other studies using various methods of splanchnic hypoperfusion. Using cardiopulmonary bypass in a porcine model, Thomassen et al. [10] showed that the pump flow had to be reduced to approximately 50% of the normal CO value to increase the colonic intramural lactate level and lactate/pyruvate ratio. In a canine model with selective restriction of aortic blood flow, a reduction in splanchnic blood flow to approximately 50% affected the intestinal CO_2_ gradient measured with tonometry [8]. In a study with selective flow reduction of SMA, the jejunal intraluminal pH started to decrease when the flow was reduced to around 50% of baseline [9]. Furthermore, a porcine study using cardiac tamponade found that anaerobic metabolism in jejunal mucosa was initiated in an interval of an aortic flow reduction between 40 and 60% of the baseline value [7].

Both methods used in the present study had a physiological component of mesenteric venous congestion, which is highly relevant when investigating the effects of cardiac dysfunction and low CO on the GI tract. Pure arterial occlusive models or models with hypovolemia do not include mesenteric venous congestion [8, 9]. Venous congestion is of importance for the circulation of the GI tract, since occlusion of the mesenteric vein produced earlier and larger effects on the systemic and regional hemodynamic and metabolism than an occlusion of the SMA [33].

There might be concerns about whether the most vulnerable part of the large GI tract has been found when detecting effects of low CO. Using intraluminal laser Doppler in postoperative cardiac surgical patients, Thorén et al. [34] showed a possible mismatch between the total splanchnic blood flow and the local mucosal perfusion. However, intraperitoneal microdialysis, with measurements of glucose, lactate, pyruvate, lactate/pyruvate ratio, and glycerol, reflects the overall intraabdominal balance between aerobic and anaerobic metabolism [35–39]. Intraluminal microdialysis detected anaerobic GI metabolism in low cardiac output, but small intestinal intraluminal insertion is not easily done in patients with closed abdomen [7]. Intraperitoneal microdialysis may be as sensitive as local intraluminal measurements in detecting regional occlusive intestinal ischemia [40], and the present data show that intraperitoneal lactate and lactate/pyruvate ratio responded with gradual increments to low CO. Thus, further exploration of intraperitoneal microdialysis in patients susceptible to low CO, and therefore GI injury, is warranted.

The present data were used to find a relationship between the reduction in CO and the blood flow in SMA. The logarithmic equation showed that CO is not proportionally distributed to SMA throughout the various levels of CO reduction. From normal CO to a reduction of 58% of the baseline value, the decrease in SMA flow is less than expected. Thereafter, a further CO reduction causes the flow in SMA to drop more steeply. This gives an indication of how the physiological regulation of the GI tract is executed in low CO states. A similar non-linear relationship between aortic and mesenteric blood flow has been shown in conscious dogs during cardiac tamponade [41]. From a physiological point of view, a combined flow and pressure threshold for preserved intestinal perfusion and metabolism would be expected, although the exact levels to target in an individual patient with various comorbidities in a clinical setting cannot be inferred from this study.

In conclusion, both cardiac tamponade and caval vein balloon inflation caused a reproducible lowering of CO and mesenteric congestion with similar GI circulatory and metabolic effects. Gastrointestinal metabolism became increasingly anaerobic when CO was reduced to 50% of the baseline value, which is considered a moderate reduction, demonstrating the potential hazard of cardiac dysfunction on the gastrointestinal tract. Anaerobic GI metabolism in low CO can be detected using intraperitoneal microdialysis, suggesting a potential use in risk patients.

## Abbreviations

CI: Cardiac index
CO: Cardiac output
CVP: Central venous pressure
DO_2_: Oxygen delivery
GI: Gastrointestinal
MAP: Mean arterial pressure
MVP: Mesenteric venous pressure
PCWP: Pulmonary capillary wedge pressure
SMA: Superior mesenteric artery
SO_2_: Oxygen saturation
VO_2_: Oxygen uptake
